# An Explainable Attention-Enhanced Deep Learning Model for Memory-Forensic Malware Detection

**DOI:** 10.3390/s26206380

**Published:** 2026-10-09

**Authors:** John Adejoh, Nsikak Pius Owoh, Moses Ashawa, Rajesh Prasad, Chibuzor Ezeamasiobi, Rajiv Ranjan Singh

**Affiliations:** 1 Department of Software Engineering, African University of Science and Technology, Abuja 900107, Nigeria; 2 Department of Computer Science, Glasgow Caledonian University, Glasgow G4 0BA, UK; 3 Department of Computer Science and Engineering, Ajay Kumar Garg Engineering College, Ghaziabad 201015, India

**Keywords:** memory forensics, malware detection, deep learning, attention mechanism, explainable artificial intelligence, SHAP, LIME

## Abstract

Memory-forensic malware detection can identify malicious activity from volatile memory and complement disk-based analysis. However, results are difficult to compare when datasets, preprocessing, model settings, and evaluation protocols differ. We propose a hybrid convolutional neural network and bidirectional gated recurrent unit (CNN–BiGRU) model with additive attention for binary malware detection on the CIC-MalMem-2022 dataset. It was compared with a one-dimensional convolutional neural network (CNN1D), bidirectional gated recurrent unit (BiGRU), bidirectional long short-term memory (BiLSTM), and Transformer models under a common protocol. All models used the same data partitions, preprocessing, optimization settings, training budget, and five training seeds. On the held-out test set of 8790 samples, the proposed model achieved 99.97% ± 0.04% accuracy, 99.97% ± 0.04% F1-score, and a Matthews correlation coefficient (MCC) of 99.93% ± 0.07%. Mean false-positive and false-negative rates (FPR and FNR) were 0.03% and 0.04%, respectively. Corrected pairwise tests did not establish statistically significant differences. SHapley Additive exPlanations (SHAP) and Local Interpretable Model-agnostic Explanations (LIME) showed a mean top-10 Jaccard overlap of 0.651 ± 0.136. SHAP required 7.96 s, compared with 35.38 s for LIME, and the proposed model had a 0.060 ms per-sample inference latency.

## 1. Introduction

Malware continues to evolve as adversaries adopt concealment, persistence, and evasion techniques. Although signature-based detection remains effective against previously characterized threats, its reliance on known patterns can limit its ability to detect obfuscated or previously unseen variants. Memory forensics provides complementary evidence because volatile memory can retain traces of malicious activity that are absent from, altered on, or deliberately obscured within persistent storage. Memory analysis can reveal running processes, loaded modules, injected code, open handles, and other indicators of runtime behavior that are difficult to observe through disk-based inspection alone. This capability is particularly important for fileless attacks, process hollowing, and in-memory payload decryption, which may leave little or no persistent evidence. However, volatile memory is lost when a system is shut down, making acquisition and analysis inherently time-sensitive. This creates a need for detection methods that can process memory data quickly and reliably during an active investigation. Prior studies have shown that memory-derived features can provide strong discriminative power for distinguishing benign and malicious activity [1,2,3,4]. Recent work has explored image-based [5,6], feature-based [7,8], and direct memory-file [9,10] representations, further demonstrating the value of memory evidence for malware detection.

In operational settings, memory acquisition and triage are often performed early in an incident-response investigation. An investigator may capture a raw memory image from a suspect host using WinPmem, DumpIt, or a hypervisor-level snapshot. The image can then be analyzed with a forensic framework such as Volatility [2]. During large-scale incidents, including ransomware outbreaks, an organization may generate dozens or hundreds of memory images within a short period. Manual examination of these images can delay containment decisions. Automated detection can reduce this burden by rapidly screening memory images while maintaining a low false-positive rate [3]. False positives consume investigative time, whereas false negatives may allow compromised systems to remain active on the network. Automated detection should therefore support both accurate screening and subsequent forensic interpretation. Memory artifacts such as process history, injected code regions, and command-line arguments can also contribute to incident reconstruction and formal reporting. Consequently, a detection system should provide explanations that investigators can interpret and, where necessary, defend. A probability score without supporting evidence is of limited forensic value.

Machine learning (ML) and deep learning (DL) methods enable the exploitation of behavioral, structural, and memory-derived features without relying exclusively on fixed signatures [4]. DL models can learn nonlinear and hierarchical representations from complex feature spaces. Convolutional neural networks (CNNs) are effective at capturing local feature interactions, whereas recurrent architectures can model dependencies across sequential representations. Attention mechanisms can further emphasize informative features within learned representations. These properties have motivated the development of hybrid architectures that combine complementary modeling capabilities [5,11,12,13]. Transformer-based methods extend this approach through self-attention mechanisms that capture long-range dependencies [14,15,16].

Despite these advances, comparing results across studies remains difficult. Reported performance can vary substantially across datasets, feature representations, preprocessing procedures, data partitioning, class balancing, optimization settings, and computational environments [4,8]. High detection performance has been reported on established benchmarks such as CIC-MalMem-2022, but differences in experimental design can make direct comparisons difficult. For example, a higher accuracy score may reflect differences in preprocessing or data partitioning rather than an architectural advantage. This issue is particularly relevant for hybrid models, where additional layers may provide only modest predictive gains while increasing model complexity or inference cost. Moreover, accuracy alone is insufficient for security applications. A practical detector should also control false positives and false negatives, operate within reasonable computational limits, and provide explanations that support analyst interpretation. A controlled evaluation under a common experimental protocol is therefore needed to isolate architectural effects from differences in experimental conditions.

Explainability is particularly important when model predictions are used to support forensic decisions. SHapley Additive exPlanations (SHAP) and Local Interpretable Model-agnostic Explanations (LIME) have been applied in cybersecurity and malware detection to identify features that influence model predictions [1,3,17]. Recent memory forensics studies have also incorporated explainability to connect automated predictions to forensic evidence [2,18,19]. However, producing an explanation does not necessarily mean that the explanation is stable, informative, or computationally practical. Explainability methods should therefore be evaluated quantitatively rather than relying solely on visual importance plots.

To address these limitations, we propose a hybrid convolutional neural network-bidirectional gated recurrent unit (CNN–BiGRU) model with additive attention for binary malware detection on the CIC-MalMem-2022 dataset. The proposed model is evaluated against a one-dimensional convolutional neural network (CNN1D), a bidirectional gated recurrent unit (BiGRU), a bidirectional long short-term memory (BiLSTM), and a Transformer encoder using a common experimental protocol. The protocol uses the same dataset, data partitions, preprocessing pipeline, optimization settings, training budget, early-stopping criteria, and checkpoint-selection rules for all architectures. The same evaluation metrics and five-seed training procedure are also applied to every model. Computational measurements are obtained under the same hardware environment. These controls are intended to reduce the influence of experimental factors that could otherwise obscure architectural differences. Prior evaluations of CIC-MalMem-2022 commonly differ in data preparation, feature representation, partitioning, class balancing, or computational settings. Such differences limit direct numerical comparison across published results. Our protocol, therefore, provides an internal standardization for the architectures evaluated in this study rather than implying that previous studies used identical conditions.

Beyond predictive performance, we examine false-positive and false-negative behavior, quantitatively compare SHAP and LIME, measure computational efficiency, and assess the contribution of the attention mechanism through ablation experiments. The study is designed to determine whether the proposed architecture provides a reproducible balance among detection performance, error rates, interpretability, and computational cost. All competing models are evaluated under the same controlled conditions. The main contributions of this study are as follows:

1. We propose a hybrid approach that combines one-dimensional convolutional feature extraction, bidirectional gated recurrent processing, and additive attention-based feature weighting for memory-forensic malware detection.
2. We perform a controlled comparison between the proposed model and the CNN1D, BiGRU, BiLSTM, and Transformer baselines using a common experimental protocol and a structured ablation study.
3. We quantitatively compare SHAP and LIME using top-feature overlap, deletion-based sensitivity, stability, and runtime on a fixed, class-balanced test subset.
4. We characterize the trade-off between predictive performance and computational efficiency using error rates, parameter count, model size, training time, inference latency, and throughput.

The rest of this paper is organized as follows. Section 2 reviews related work on memory-based malware detection and explainable artificial intelligence. Section 3 describes the dataset, preprocessing procedures, proposed methodology, and experimental protocol. Section 4 presents the experimental and explainability results. Section 5 discusses the findings, limitations, and practical implications.

## 2. Related Work

Malware detection research has increasingly incorporated memory-forensic analysis to identify malicious activity that may evade traditional disk-based scanning. Existing studies span classical machine learning, deep learning, hybrid neural networks, Transformer-based models, and explainable artificial intelligence. This section reviews these approaches with emphasis on their reported performance, architectural characteristics, explainability, and computational requirements.

### 2.1. Classical Machine Learning for Memory-Forensic Malware Detection

Early studies showed that memory-derived features can support effective malware detection without complex neural architectures. Dener et al. [7] evaluated several machine learning and deep learning algorithms on CIC-MalMem-2022 and reported 99.97% accuracy with Logistic Regression. This result demonstrates that high detection performance can be achieved using relatively simple classifiers when informative memory features are available. Other studies have extended classical machine learning approaches to more challenging settings. Cevallos-Salas et al. [20] investigated obfuscated privacy malware using memory-dumping analysis and evaluated models for both binary and multiclass classification. Hossain and Islam [21] combined class balancing and feature selection procedures with machine learning models and reported detection performance above 99%. Imamverdiyev and Baghirov [22] further incorporated SHAP and LIME into an ensemble framework for detecting obfuscated malware. These studies establish two important points. First, memory-derived features can provide strong discrimination between benign and malicious samples. Second, high predictive performance does not necessarily indicate methodological superiority, as reported results are influenced by preprocessing, feature selection, class balancing, and evaluation protocols. Computational measures such as training time, inference latency, and memory consumption are also inconsistently reported [7,20,21,22]. These differences make direct comparison across studies difficult and motivate controlled evaluations using common experimental conditions.

### 2.2. Deep Learning and Hybrid Neural Architectures for Memory-Based Malware Detection

Deep learning methods extend conventional machine learning by learning nonlinear representations from memory-derived features or memory-based representations. Mezina and Burget [11] proposed a dilated convolutional network for obfuscated malware detection and reported 99.00% accuracy on the CIC-MalMem-2022 dataset. Shafin et al. [12] developed a compact CNN–BiLSTM architecture for obfuscated memory malware, with particular attention to processing speed and model compactness in resource-constrained environments. Their results suggest that combining convolutional and recurrent representations can provide an effective balance between detection capability and computational requirements. Other studies have explored more complex architectures and representations. Naeem et al. [23] proposed a deep-stacked CNN ensemble based on process-level volatile memory forensics and incorporated explainability into the detection pipeline. Liu et al. [9] developed MRm-DLDet by combining ResNet-18 feature extraction with an attention-augmented GRU classifier for memory-resident malware detection. Zhang et al. [10] applied a one-dimensional CNN to executable fragments recovered from memory and reported 97.48% accuracy, showing that memory representations beyond pre-extracted feature vectors can also support malware classification. Rai et al. [24] subsequently proposed a deep learning framework for fileless malware detection using volatile-memory forensics. The literature therefore supports the use of convolutional, recurrent, and attention-based components for memory-oriented malware detection. However, closely related CNN-recurrent and attention-recurrent architectures have already been reported [9,23]. The contribution of the present study, therefore, does not rest solely on the combination of these components. Instead, the study examines a CNN–BiGRU architecture with additive attention under a common experimental protocol. The analysis focuses on the balance among predictive performance, detection errors, and computational cost relative to the CNN, BiGRU, BiLSTM, and Transformer baselines.

### 2.3. Attention and Transformer-Based Architectures

Attention mechanisms and Transformer architectures have gained increasing interest in malware analysis because they can model relationships among distributed or heterogeneous security features. Alrawashdeh [14] investigated a textual-attention Transformer for memory reverse engineering and malware behavior reconstruction. The study demonstrated the potential of self-attention to capture relationships among memory-derived representations that may be difficult to model with conventional local feature extraction alone. Transformer-based approaches have also been applied beyond direct memory-forensic analysis. Ashawa et al. [6] examined Transformer-based convolutional architectures for image-based malware classification. At the same time, Qian and Cong [25] proposed a Transformer-based malware identification model that combines channel attention with a CNN–LSTM classifier for API-call sequences. Although these studies use different input representations from CIC-MalMem-2022, they reflect a broader trend toward combining convolutional, recurrent, and attention mechanisms in malware analysis.

Attention has also been incorporated into memory-oriented detection systems. Liu et al. [9] employed an attention–GRU architecture for memory-resident malware detection, while recent work has investigated attention-driven and interpretable memory-artifact detection frameworks [19]. These studies support the use of attention for both representation learning and feature attribution. However, their differing datasets, input representations, preprocessing procedures, and evaluation protocols make it difficult to determine whether the additional complexity of an attention-based hybrid model provides sufficient benefit over simpler architectures. The present study addresses this issue by conducting a controlled comparison among CNN, BiGRU, BiLSTM, Transformer, and the proposed CNN–BiGRU–Attention architecture. The objective is to evaluate the architectural differences under the same data and experimental conditions rather than infer superiority from results obtained under different protocols.

### 2.4. Explainability, Computational Efficiency, and Generalization

In forensic malware detection, predictive performance alone may not be sufficient, as model outputs can inform subsequent investigation and incident-response decisions. Explainability methods can identify memory-derived features associated with a prediction and help analysts interpret the basis of a classification. Imamverdiyev and Baghirov [22] incorporated both SHAP and LIME into a memory-based malware detection framework, while Naeem et al. [23] integrated explainability into a deep memory-forensic pipeline. More recent work has combined malware detection with SHAP-based interpretation and attention-based feature analysis [19]. However, the inclusion of an XAI method does not by itself establish that the resulting explanations are stable, informative, or computationally practical. Hermosilla et al. [26] compared SHAP and LIME in forensic intrusion-detection models and emphasized the importance of evaluating explanation behavior rather than presenting visualizations alone. Recent explainability research has also explored natural-language generation and prompt-based methods for producing explanations that are easier for end users to understand. Li et al. [27] investigated personalized prompt learning for explainable recommendation, demonstrating how prompt-based methods can support the generation of user-understandable explanations. Breve et al. [28] extended this line of work to security by generating natural-language justifications for security and privacy risks associated with automation rules. These approaches provide a complementary form of explainability in which model outputs are communicated as human-readable justifications rather than feature-attribution scores. In the present study, SHAP and LIME are used for quantitative feature-level attribution, while natural-language generation is considered a complementary direction for future work.

Computational efficiency is similarly important for practical deployment. Shafin et al. [12] emphasized model compactness and processing speed, while other studies have reported computational measures such as detection or explanation latency [10,25]. Recent memory forensics research has also considered efficiency across memory extraction, machine learning, and explainability stages [14]. However, these measurements are generally obtained under different hardware configurations, datasets, input representations, and experimental protocols. Consequently, comparisons across studies may not reflect architectural differences alone. Generalization also remains an important consideration. CIC-MalMem-2022 provides a controlled benchmark for comparative experimentation, but performance on a single dataset should not be interpreted as evidence of broad real-world effectiveness. Prior memory-forensics research has shown that domain and environmental differences can influence model performance [18]. The present study, therefore, treats CIC-MalMem-2022 as a controlled benchmark for architectural comparison and limits its conclusions to the evaluated experimental conditions.

### 2.5. Research Gap and Positioning of the Present Study

Three gaps motivate the present study. First, architectural comparisons in memory-forensic malware detection are difficult because previous studies often use different preprocessing procedures, feature representations, data partitions, class-balancing strategies, and computational environments. A common protocol is therefore needed to reduce these sources of variation. Second, predictive performance and computational cost are not consistently evaluated together. High accuracy may coexist with false positives, false negatives, increased parameter count, or higher inference cost. These factors are relevant when a detector is intended for practical forensic screening. Third, explainability is often presented through feature-importance visualizations without systematic comparison of attribution agreement, sensitivity, stability, and runtime. Evaluating SHAP and LIME on the same trained model and test subset provides a controlled basis for examining these properties.

The present study addresses these gaps using a CNN–BiGRU model with additive attention on the CIC-MalMem-2022 dataset. The proposed model is compared with CNN1D, BiGRU, BiLSTM, and Transformer baselines under matched experimental conditions. Evaluation covers detection metrics, FPR and FNR, computational efficiency, architectural ablation, and quantitative SHAP–LIME analysis. CIC-MalMem-2022 is treated as a controlled benchmark for architectural comparison. The findings are not assumed to establish generalization to other memory-forensics datasets or acquisition environments. This gap motivates the systematic error-rate analysis (Section 4.3) and computational-efficiency evaluation (Section 4.7) reported for the proposed model, as further shown in Table 1.

## 3. Materials and Methods

This study develops and evaluates a hybrid deep learning model for malware detection using memory-forensic features. The proposed model is compared with four baseline architectures under a common experimental protocol. The experimental procedure comprised data preparation, model training, threshold selection, explainability analysis, and performance evaluation. The same data partitions, preprocessing procedure, optimizer, training budget, stopping criterion, and evaluation protocol were used for all five models. The study also includes a five-condition ablation analysis, validation-based threshold selection, a quantitative SHAP–LIME comparison, and statistical analysis across five independent training seeds. This controlled design reduces variation arising from differences in experimental conditions and enables a consistent comparison of the model architectures.

### 3.1. Dataset

The experiments use the CIC-MalMem-2022 memory-forensics dataset, developed by the Canadian Institute for Cybersecurity at the University of New Brunswick and introduced by Carrier et al. [29]. The dataset was generated from volatile memory snapshots collected in a controlled Windows environment. Clean sessions were alternated with sessions in which malware samples obtained from VirusTotal were executed. Memory artifacts were then extracted using the VolMemLyzer Volatility 2, feature-extraction tool.

VolMemLyzer provides features from nine categories: process-list statistics (pslist), loaded-DLL statistics (dlllist), open-handle statistics (handles), module-loading information (ldrmodules), and memory-injection indicators (malfind). The remaining categories are process-hiding cross-checks (psxview), kernel-module statistics (modules), Windows-service statistics (svcscan), and kernel-callback statistics (callbacks). These categories capture complementary characteristics of processes, modules, handles, services, and other runtime artifacts observed in memory.

The dataset contains 58,596 labeled records, comprising 29,298 benign and 29,298 malicious samples. Before feature reduction, each sample contains 55 raw numerical features. The malicious samples represent three broad malware families: ransomware, spyware, and Trojan horses. These families comprise 15 subtypes and exhibit diverse malware behaviors. The subtype distributions are 9791 ransomware samples, 10,020 spyware samples, and 9487 Trojan samples.

The released dataset also contains the class and category fields. The Class field provides the binary target label, whereas category identifies the malware subtype. The category was retained as metadata but was not used as an input feature. The classification task was therefore formulated as a binary decision between benign and malicious samples. This formulation focuses the analysis on detection performance, error behavior, interpretability, and computational efficiency rather than malware-family attribution.

Memory snapshots were captured in debug mode to prevent the acquisition process itself from introducing additional processes into the memory image. Thus, the extracted features represent the observed system state at the time of acquisition. The dataset is publicly available through the Canadian Institute for Cybersecurity [29].

### 3.2. Data Preparation

Preprocessing was performed in a fixed sequence. First, the target and metadata fields were separated from the feature matrix. The Filename identifier, non-numeric columns, constant features, and exact duplicate feature columns were excluded. This procedure reduced the feature representation to 52 numerical variables per sample. Second, exact duplicate feature vectors were identified using a row-wise hash computed from the 52 retained numerical features, independently of the class label. Duplicate feature groups were kept together and assigned to a single partition before data splitting. This group-based approach prevented identical feature vectors from appearing across the training, validation, and test sets. A dataset audit identified 569 rows belonging to duplicate hash groups. After splitting, verification confirmed that no duplicate hashes crossed the train–validation, train–test, or validation–test boundaries. All 569 duplicate rows were contained in the training set.

After duplicate groups were identified, the data were divided into training, validation, and test sets using stratified sampling at a 70:15:15 ratio. The stratification preserved the benign–malicious class balance across the partitions. A fixed split seed of 42 was used for all models. Five training seeds (42, 52, 62, 72, and 82) were used to assess sensitivity to random initialization and batch ordering. Infinite values were treated as missing and imputed using the median of the feature calculated from the training partition only. Min-max scaling parameters were also estimated from the training partition and applied unchanged to the validation and test sets. The validation set was used for early stopping, checkpoint selection, and threshold selection, whereas the test set was reserved for final evaluation. Min-max normalization was applied as follows:

(1) $$x^{\prime }=\frac{x-{min}_{train}}{{max}_{train}-{min}_{train}}$$

where x is the original feature value, $x^{\prime }$ is the normalized feature value ${min}_{train}$ and ${max}_{train}$ are the minimum and maximum values of the corresponding feature calculated from the training partition. The same training-derived values were applied unchanged to the validation and test partitions. Therefore, no preprocessing statistic was estimated from the evaluation data.

### 3.3. Proposed Model Architecture

**Figure 1:**
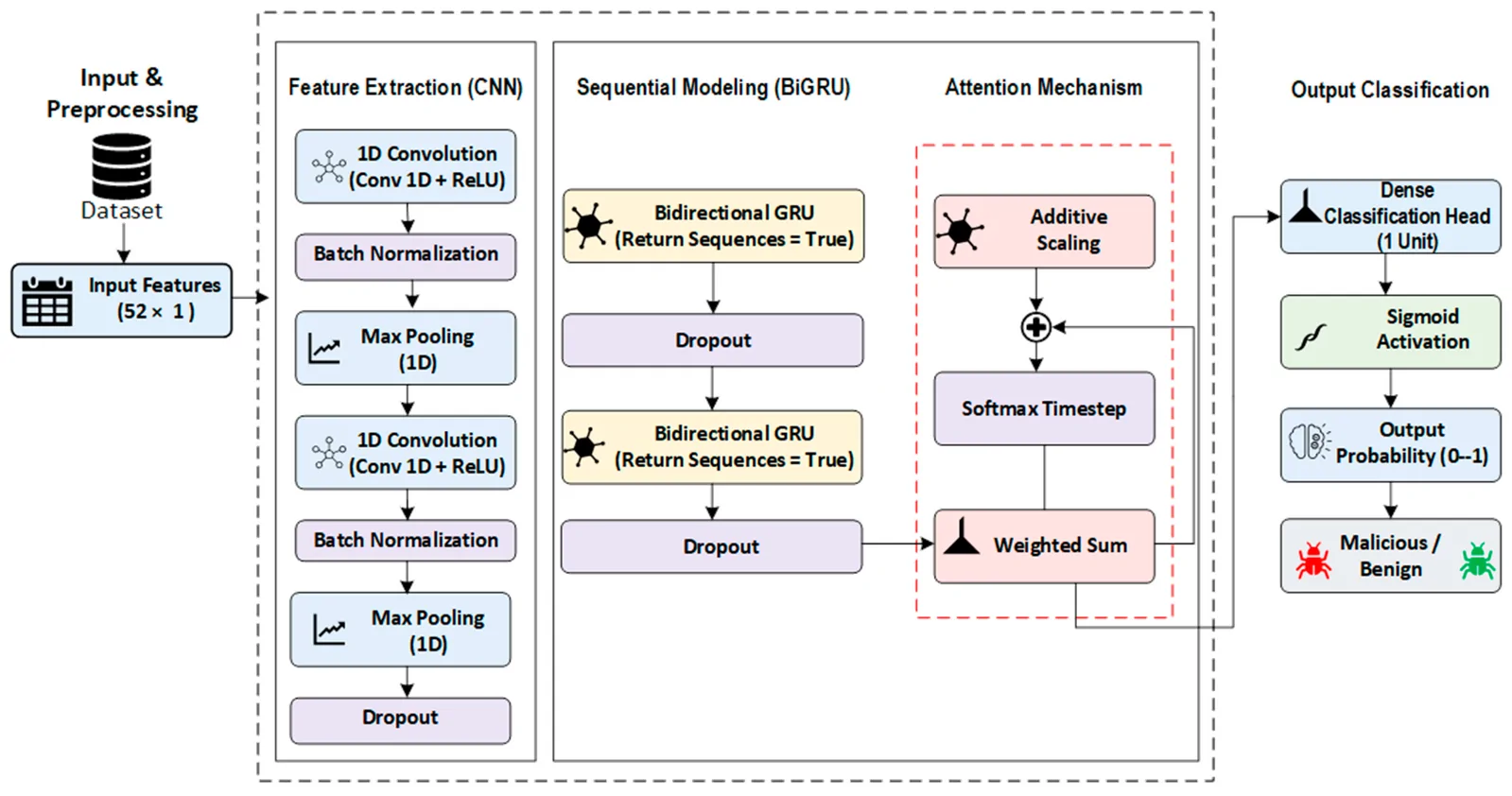
Model architecture.

The proposed model combines one-dimensional convolution, bidirectional recurrence, and attention. The architecture first extracts local patterns from the memory-forensic feature sequence and then models contextual relationships among the resulting representations. This design follows the general principle of combining convolutional and recurrent representations for structured or sequential malware data [8,17]. Each sample is represented as a sequence of 52 features with a single input channel. The features retain their original column order, and no domain-specific reordering is applied. Therefore, the model learns any useful positional relationships directly from the training data. Because convolutional and recurrent operations depend on input order, sensitivity to alternative feature orderings remains a potential limitation of the architecture and is not evaluated in the present study. Figure 1 presents the proposed architecture.

The first convolutional block uses 64 filters with a kernel size of 3, followed by batch normalization and max-pooling. The second block uses 128 filters with a kernel size of 5 and applies the same normalization and pooling operations. The convolutional layers capture local interactions between adjacent input positions and reduce the sequence length prior to recurrent processing. The resulting sequence is processed by a bidirectional GRU with 128 units in each direction. The forward and backward hidden states are concatenated at each timestep, producing a 256-dimensional representation:

(2) $$H=\left[h_{1},h_{2},\ldots ,h_{T}\right],\;h_{t}\in R^{256}$$

where H denotes the sequence of BiGRU hidden-state representations, T is the number of resulting timesteps, and $h_{t}\in R^{256}$ is the concatenated forward and backward hidden state at timestep t.

(3) $$e_{t}=v^{⊤}tanh\left({WH}_{t}+b\right)$$

where $e_{t}$ denotes the attention score at timestep t, $H_{t}$ is the BiGRU hidden-state representation at timestep t, W and v are trainable attention parameters, b is a trainable bias term, and $tanh\left(\cdot \right)$ is the nonlinear activation function used in the attention projection.

(4) $$\alpha_{t}=\frac{exp\left(e_{t}\right)}{\sum_{j}exp\left(e_{j}\right)}$$

$\alpha_{t}$ denotes the normalized attention weight assigned to timestep t, $e_{t}$ is its corresponding attention score, and the denominator is the sum of the exponentiated attention scores over all timesteps.

(5) $$c=\sum_{t}\alpha_{t}H_{t}$$

where c denotes the attention-weighted context representation, $H_{t}$ is the BiGRU hidden-state representation at timestep t, and $\alpha_{t}$ is the corresponding normalized attention weight. The resulting context vector summarizes the recurrent sequence while assigning greater weight to more informative timesteps. Each timestep represents a receptive field produced by the preceding convolution and pooling operations. Therefore, the attention weights should not be interpreted as direct, one-to-one feature importance for the original 52 variables. Feature-level interpretation is instead obtained from the SHAP and LIME analyses described in Section 3.6. The context vector is passed through a dropout layer with a rate of 0.40, followed by a fully connected layer with 128 ReLU-activated units. A second dropout layer with the same rate is applied before the final sigmoid output layer. The output represents the predicted probability of the malicious class. The complete model contains 306,305 parameters. The forward computation is summarized in Algorithm 1.
**Algorithm 1.** Forward pass of the proposed model
**Input:** x ∈ ℝ^d×1^—preprocessed, min–max scaled feature sequence, d = 52; Trained weights: CNN filters, BiGRU weights, attention parameters (W, b, v), classification-head weights
**Output:** ŷ ∈ [0, 1]—predicted probability of the malicious class1h^(1)^ ← ReLU(BatchNorm(Conv1D_64_,_k=3_(x)))2p^(1)^ ← MaxPool_2_(h^(1)^)3h^(2)^ ← ReLU(BatchNorm(Conv1D_128_,_k=5_(p^(1)^)))4p^(2)^ ← MaxPool_2_(h^(2)^)5**for** t = 1 to T **do:**6    h_forward[t] ← GRU_128_(p^(2)^[t], h_forward[t−1])7**end for**8**for** t = T downto 1 **do:**9   h_backward[t] ← GRU_128_(p^(2)^[t], h_backward[t+1])10**end for**11H ← [h_forward[t]; h_backward[t]]_t=1_,…,_t_12**for** t = 1 to T **do:**13  e[t] ← v^T^·tanh(W·H[t] + b)14**end for**15α ← softmax(e)16c ← Σ_t=1_^T^ α[t]·H[t]17z ← ReLU(Dense_128_(Dropout_0.40_(c)))18ŷ ← σ(Dense_1_(Dropout_0.40_(z)))19**return** ŷ

### 3.4. Baseline Architectures and Training Protocol

Four baseline architectures were used for comparison: CNN1D, BiGRU, BiLSTM, and a Transformer encoder. CNN1D retained the two convolutional blocks used in the proposed architecture and replaced the recurrent and attention stages with global average pooling. BiGRU and BiLSTM used a single bidirectional GRU or LSTM layer with 128 units per direction and operated directly on the input sequence. Their hidden-state sequences were reduced using global average pooling before entering the shared classification head. The Transformer baseline used a dense projection to 128 dimensions, followed by fixed, non-trainable positional encoding. It then applied a multi-head self-attention block with four heads and a key dimension of 32, followed by a feed-forward sublayer with a hidden dimension of 128. Residual connections and layer normalization were applied around the Transformer components. The output sequence was then reduced using global average pooling before the classification head. A separate five-condition ablation study was performed to isolate the contributions of the architectural components.

The ablation conditions were CNN1D, CNN–BiGRU, CNN–Attention, BiGRU–Attention, and the complete proposed model. These conditions were analyzed separately from the four-baseline comparison. All models were trained using Adam with a learning rate of $1\times 10^{-3}$, binary cross-entropy loss, and a batch size of 64. Training continued for up to 50 epochs. Early stopping monitored validation loss with a patience of 10 epochs, and the best validation weights were restored. Each architecture was trained using the five predefined random seeds. Python 3.13, NumPy 2.5, and TensorFlow 2.9 random states were fixed, and TensorFlow deterministic operations were enabled. Training states were persisted using the Keras BackupAndRestore mechanism to support recovery from runtime interruptions. The synthetic minority over-sampling technique (SMOTE) and class weighting were available in the experimental pipeline but were disabled for the reported results because the dataset was approximately class-balanced.

### 3.5. Evaluation Protocol

Model performance was evaluated on the held-out test partition using accuracy, precision, recall, F1-score, balanced accuracy, Matthews correlation coefficient (MCC), ROC-AUC, specificity, false-positive rate (FPR), and false-negative rate (FNR). ROC-AUC was calculated as the area under the receiver operating characteristic curve across decision thresholds. Because malware detection requires consideration of both false positives and false negatives, the default threshold of 0.50 was not assumed to be optimal. Candidate thresholds were evaluated using validation-set predictions only. The operating threshold was selected according to:

(6) $$\left.\left.\left.t^{*}=argt\frac{min}{t}\left.[\right.FPR_{val}\left.(\right.t)\right.+FNR_{val}\left.(\right.t)\right.]\right.$$

where t* denotes the selected decision threshold, t represents a candidate threshold, and FPRval(t) and FNRval(t) denote the false-positive and false-negative rates calculated on the validation set at threshold t. The selected threshold was then applied once to the test predictions. Thus, no test-set information was used during threshold selection. The primary comparison between the proposed model and the four baseline architectures used paired one-sided Wilcoxon signed-rank tests across the five matched training seeds. The tests were conducted separately for default-threshold F1-score, false-positive rate (FPR), false-negative rate (FNR), and inference latency. Results across seeds are reported as mean ± standard deviation. Because four pairwise comparisons were performed for each metric in the main baseline analysis, the Holm–Bonferroni procedure was applied within each metric-specific family to control for multiple comparisons. No additional correction was applied across the different metrics. The same paired one-sided Wilcoxon signed-rank framework was used for the F1-score comparisons in the ablation analysis. The four ablation variants were compared with the complete proposed architecture, and the resulting F1-score *p*-values were adjusted using the Holm–Bonferroni procedure across the four pairwise comparisons. Given that only five paired observations were available for each comparison, the statistical analyses were treated as exploratory evidence of directional differences rather than as confirmatory hypothesis tests.

### 3.6. Explainability and Computational Efficiency Evaluation

SHAP and LIME were used to evaluate model interpretability. The analysis was performed on the same fixed subset of 100 test instances, comprising 50 benign and 50 malicious samples. SHAP values were generated using GradientExplainer with a fixed background set of 100 randomly selected training instances. The resulting values quantify the contribution of individual features relative to the background distribution. LIMEs were generated for the same test instances using 1000 perturbed neighbors per sample. The perturbations were weighted according to their proximity to the original instance, and a local linear surrogate model was fitted without discretizing the continuous features. The two explanation methods were compared using four criteria. Agreement was measured using the Jaccard overlap between the top-ranked feature sets. Fidelity was evaluated using a deletion test. The top-attributed features were replaced with their training-set median values, and the resulting change in the predicted probability of maliciousness was measured. Stability was assessed by repeatedly resampling the explanation procedure and comparing the resulting feature rankings. Computational cost was measured using both total runtime and average runtime per explained instance.

The top-10 feature sets were used for the agreement and stability analyses, and stability was assessed over resampling repetitions. Computational efficiency was evaluated under identical hardware conditions for all architectures. Training time, inference latency, and throughput were recorded for each model. For inference measurements, the first three passes over the test set were discarded as warm-up runs. The remaining timed passes were used to calculate per-sample latency and throughput. Parameter count and on-disk model size were also recorded. Central processing unit (CPU) memory usage after training and, where available, peak graphics processing unit (GPU) memory usage were measured to characterize deployment requirements. These measurements complement the detection results by describing the computational cost associated with each architecture. Finally, the complete experimental configuration was stored in a machine-readable manifest. The manifest included hyperparameters, random seeds, partition ratios, threshold-selection rules, and the SHA-256 hash of the dataset file. Per-run artifacts were organized by model and seed, including model checkpoints, training histories, predictions, confusion matrices, and SHAP/LIME value tables.

## 4. Results

All five architectures were evaluated on the held-out test partition using the experimental protocol described in Section 3. Unless otherwise stated, results are reported at the default classification threshold of 0.50 as the mean ± standard deviation across five independent training seeds.

### 4.1. Training and Validation Performance

**Figure 2:**
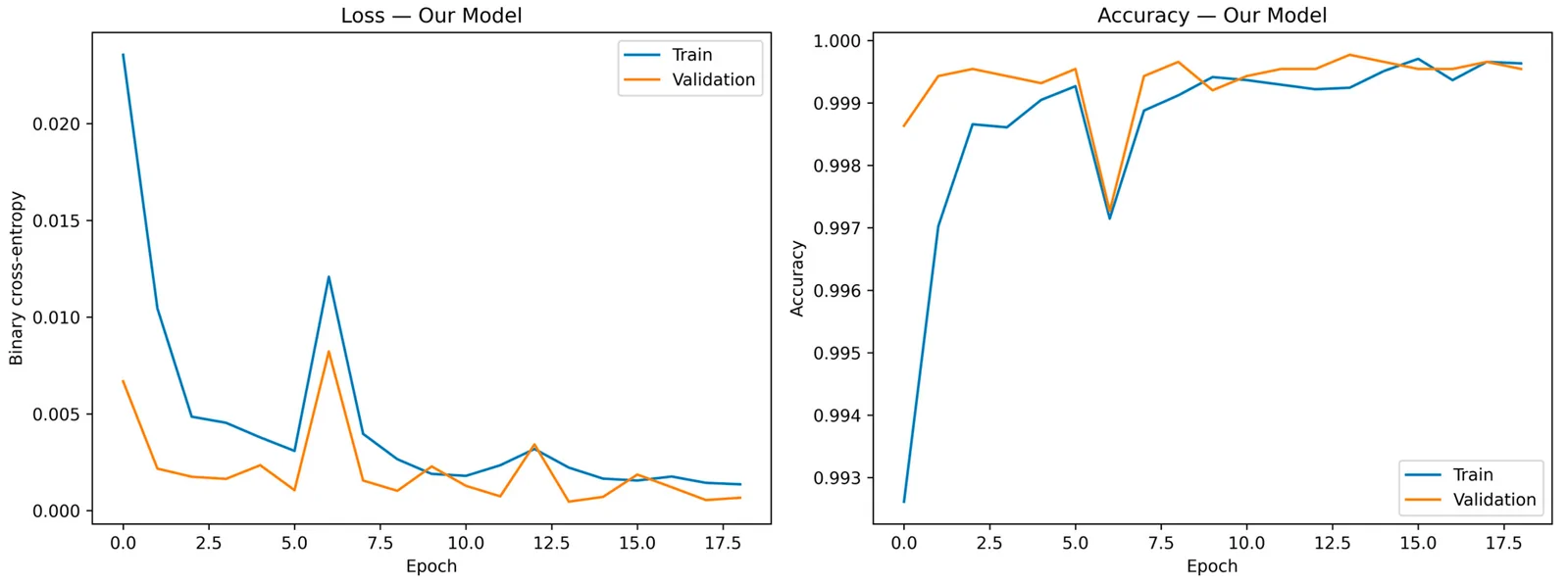

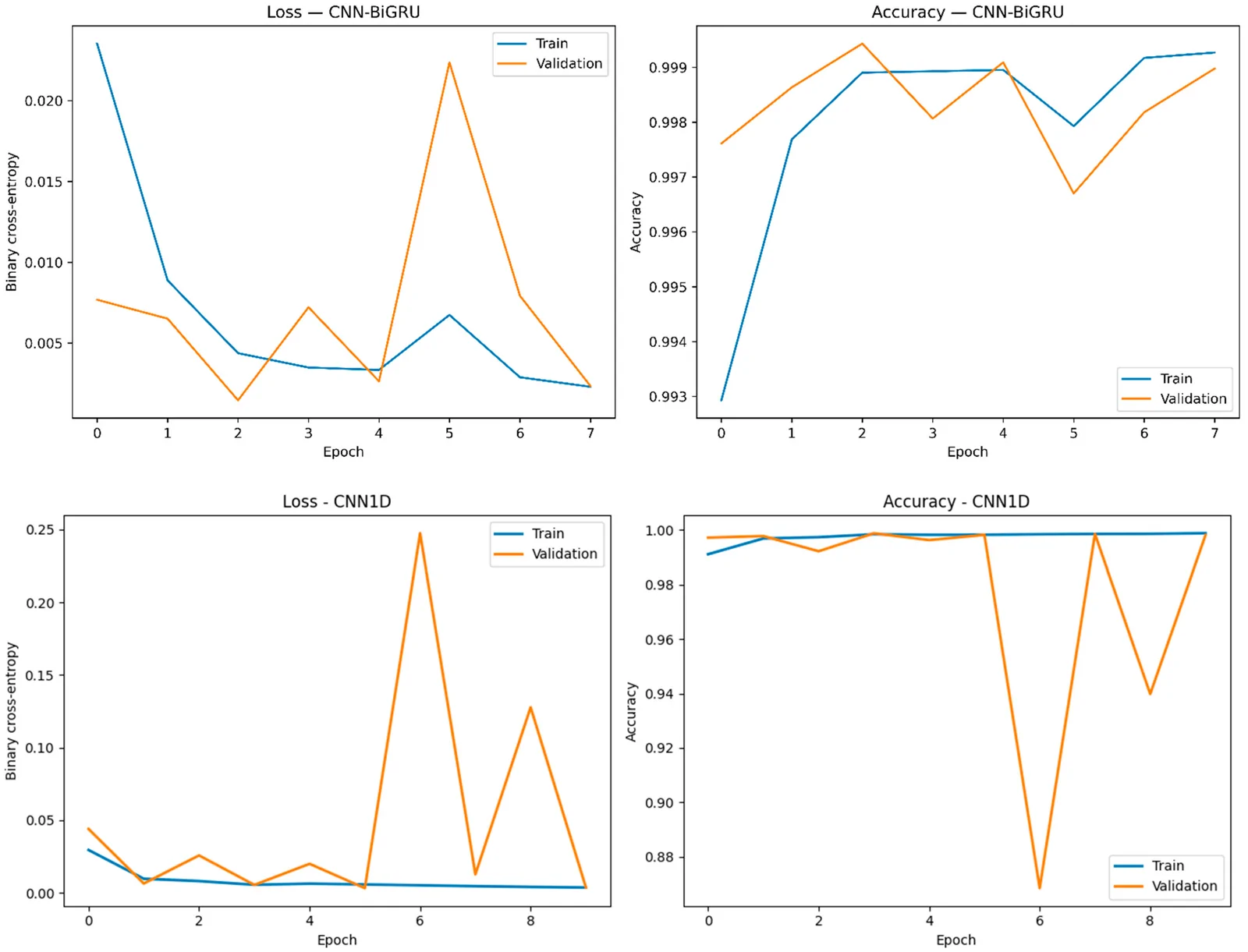
Training and validation loss and accuracy for the selected architectures.

Figure 2 presents the training and validation loss and accuracy curves for the selected architectures. The proposed model achieves a validation accuracy close to 0.999 in the early training epochs and maintains a small gap between the training and validation curves. Its loss also decreases rapidly and remains low during subsequent epochs. CNN–BiGRU and CNN1D show greater short-term variation in validation loss and accuracy. Overall, the curves indicate stable convergence under the adopted training protocol. The strong validation performance is consistent with the near-ceiling test results reported in Table 2.

### 4.2. Detection Performance

Table 2 summarizes the default-threshold performance of the five architectures. All models achieve accuracy and F1-scores above 0.99, with relatively small differences among the highest-performing models.

The proposed model recorded the highest observed accuracy, precision, F1-score, and MCC. The corresponding values were 0.9997 ± 0.0004, 0.9997 ± 0.0003, 0.9997 ± 0.0004, and 0.9993 ± 0.0007, respectively. CNN1D has a slightly higher recall point estimate of 0.9997 ± 0.0004, compared with 0.9996 ± 0.0006 for the proposed model. The corresponding FNR values are also very close. The proposed model, CNN1D, and Transformer each achieve an ROC-AUC of 1.0000 in the reported results. The small standard deviations across seeds indicate limited variation in the measured performance under the fixed experimental protocol. The absolute F1-score differences are also small, with increases of 0.0009, 0.0028, and 0.0079 relative to CNN1D, BiGRU, and BiLSTM, respectively. BiLSTM records the lowest F1-score among the five primary architectures at 0.9918 ± 0.0004. CNN1D and Transformer achieve F1-scores of 0.9988 ± 0.0006 and 0.9974 ± 0.0007, respectively. These results show that all five architectures perform strongly on the benchmark and that the performance differences among the strongest models are small.

**Figure 3:**
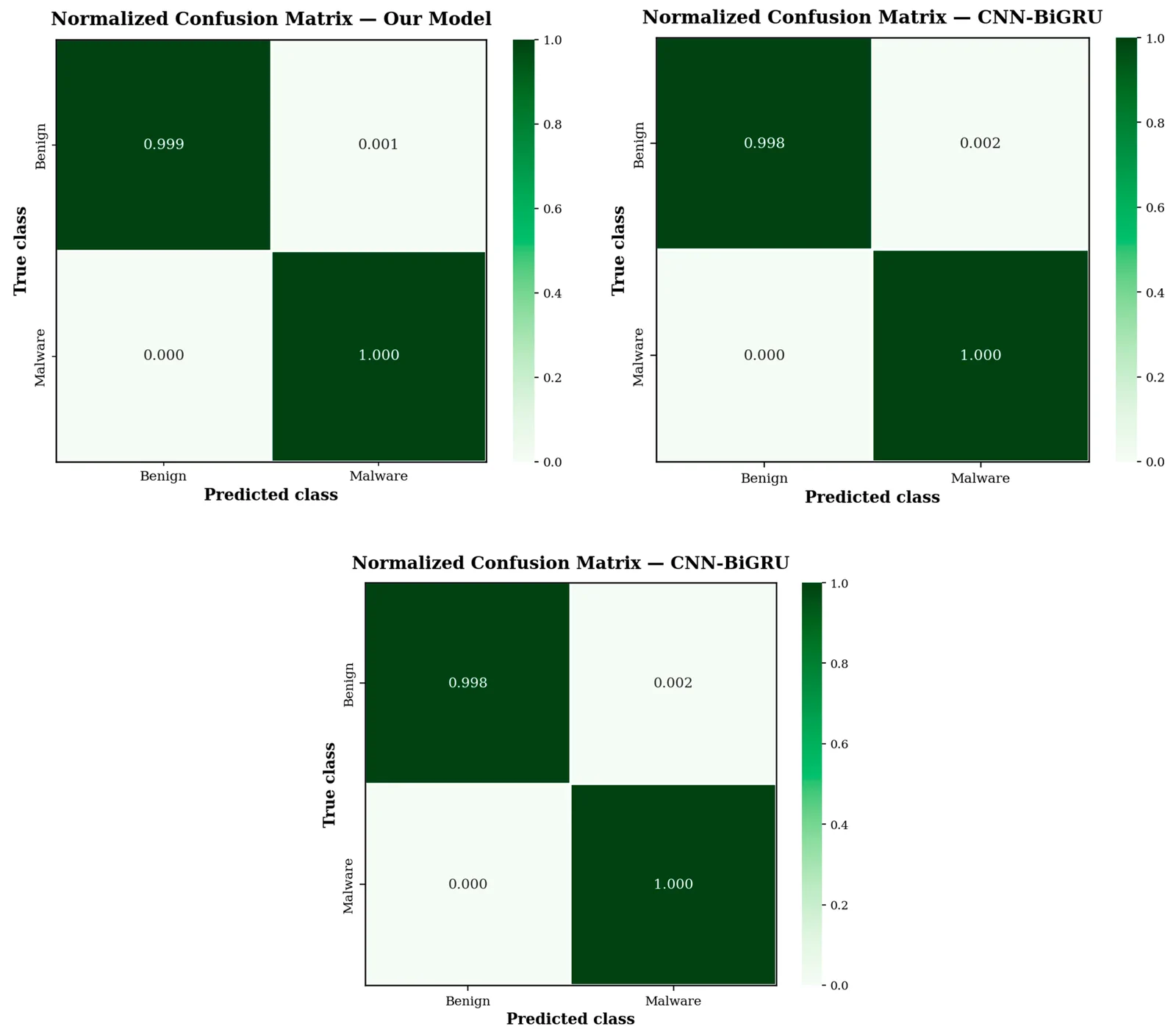
Normalized confusion matrices for the selected architectures.

Figure 3 presents normalized confusion matrices for the selected architectures. The proposed model correctly classifies nearly all benign and malicious samples, with only a small number of residual errors. Similar near-diagonal patterns are observed for the comparison models.

**Figure 4:**
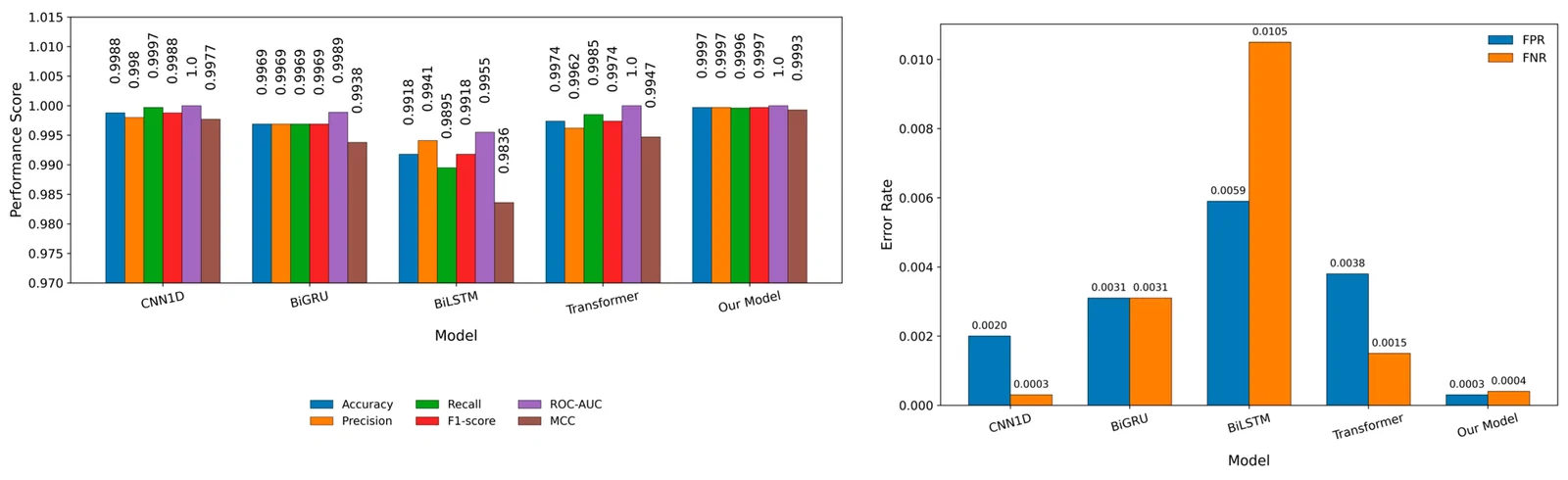
Comparative performance of the evaluated architectures.

Figure 4 provides a visual comparison of the reported performance metrics. The results show that the models operate near the benchmark’s upper performance range, with only small differences among the strongest architectures.

**Figure 5:**
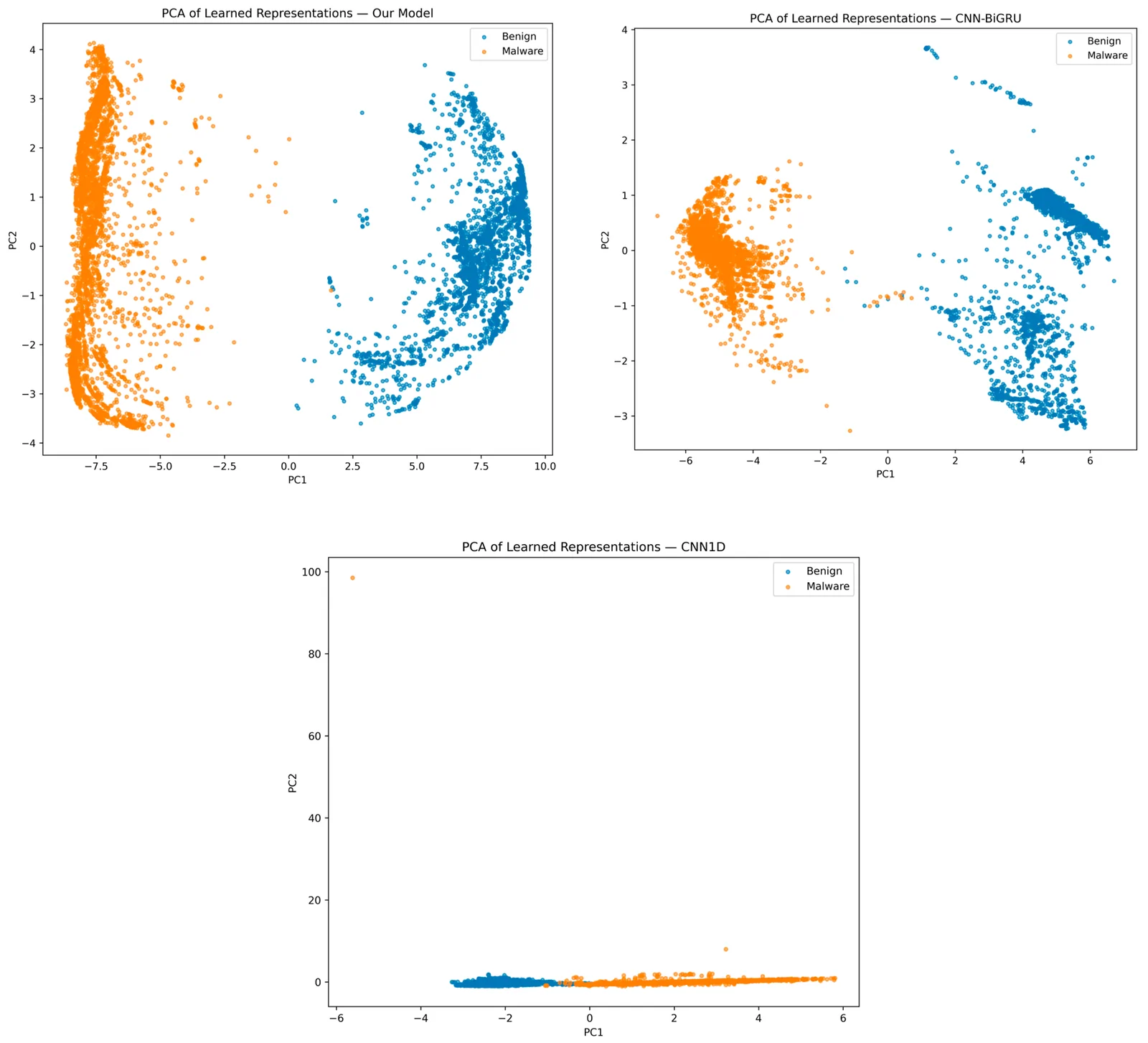
Two-dimensional PCA projections of learned representations.

To provide a qualitative view of the learned representations, two-dimensional principal component analysis (PCA) projections were generated for the proposed model, CNN–BiGRU, and CNN1D ablation, as shown in Figure 5. Each panel projects the learned representation onto the first two principal components, with points colored by benign or malicious class. The projections provide a visual indication of how the representations are distributed by class before the final classification output. The proposed model and CNN–BiGRU show visually separated class distributions with limited overlap, whereas the CNN1D representation is more compressed along the second principal component. These differences should be interpreted as qualitative characteristics of the projected representations rather than as direct measures of classification performance. PCA reduces the original representation to two dimensions and may therefore omit information retained in higher-dimensional components. The visual patterns are therefore considered alongside the quantitative detection results in Table 2 and Table 5.

### 4.3. Decision-Threshold and FPR/FNR Analysis

Table 3 compares the default threshold with the validation-selected threshold for the proposed model. The selected thresholds range from 0.11 to 0.51 across the five seeds. Both FPR and FNR remain close to zero at the evaluated operating points.

**Figure 6:**
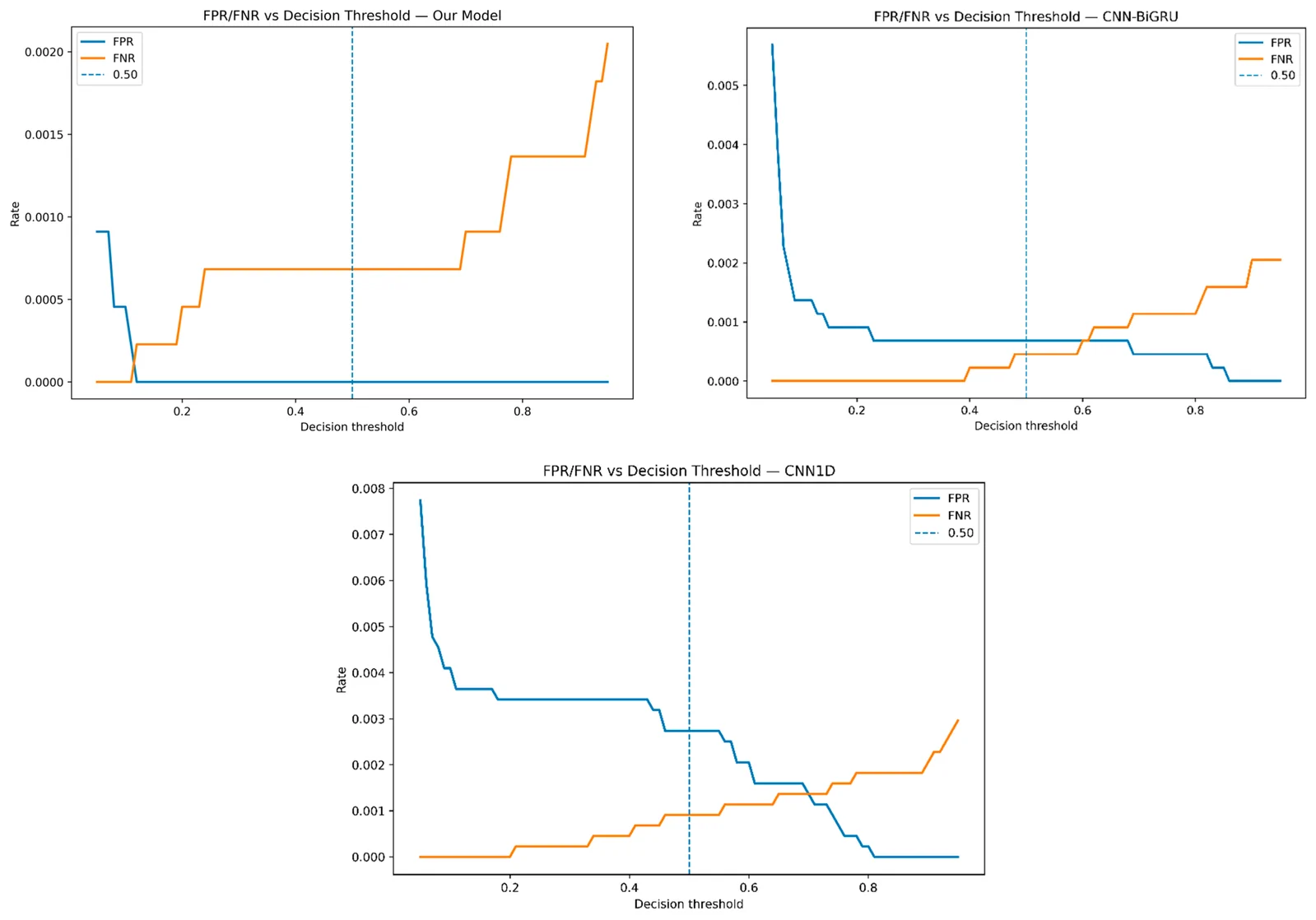
FPR and FNR as functions of the decision threshold for the evaluated models.

Figure 6 shows the variation in FPR and FNR over the evaluated decision thresholds. FPR decreases as the decision threshold increases, whereas FNR remains low across a broad intermediate range before increasing at higher thresholds. The selected thresholds fall within this low-error region. The variation in selected thresholds is consistent with a relatively flat validation objective over part of the threshold range. Importantly, the threshold was selected using validation predictions only and was then applied to the test set without further adjustment.

### 4.4. Statistical Analysis

Table 4 presents the paired comparisons between the proposed model and the four baseline architectures across the five matched seeds. The proposed model has numerically higher F1-scores than all four baselines. These numerical differences should be interpreted with the Holm–Bonferroni-adjusted *p*-values in Table 4. The FPR and FNR comparisons also show small absolute differences. The latency comparisons show differences in both directions, with no clear separation between the models under the reported five-seed experiment. Given the small number of paired observations, the statistical tests are best interpreted as exploratory evidence of differences between architectures. The numerical results in Table 2 should therefore be considered together with the mean paired differences and corrected *p*-values in Table 4.

**Table 4:** One-sided paired Wilcoxon signed-rank comparisons between the proposed model and the baseline architectures.

Comparator	Metric	Mean Paired Difference	Raw *p*	Holm-Corrected *p*
BiGRU	F1-score	+0.0028	0.0313	0.1252
BiLSTM	F1-score	+0.0079	0.0313	0.1252
CNN1D	F1-score	+0.0008	0.0625	0.1252
Transformer	F1-score	+0.0023	0.0313	0.1252
BiGRU	FPR	−0.0028	0.0625	0.25
BiLSTM	FPR	−0.0056	0.0625	0.25
CNN1D	FPR	−0.0017	0.0625	0.25
Transformer	FPR	−0.0035	0.0625	0.25
BIGRU	FNR	−0.002730	0.0625	0.25
BiLSTM	FNR	−0.010102	0.0625	0.25
CNN1D	FNR	+0.000091	1.0000	1.00
Transformer	FNR	−0.001092	0.1250	0.25
BiGRU	Inference latency	−0.012698	0.0625	0.25
BiLSTM	Inference latency	−0.008304	0.0625	0.25
CNN1D	Inference latency	+0.010260	0.1875	0.375
Transformer	Inference latency	+0.004370	0.0625	0.625

**Figure 7:**
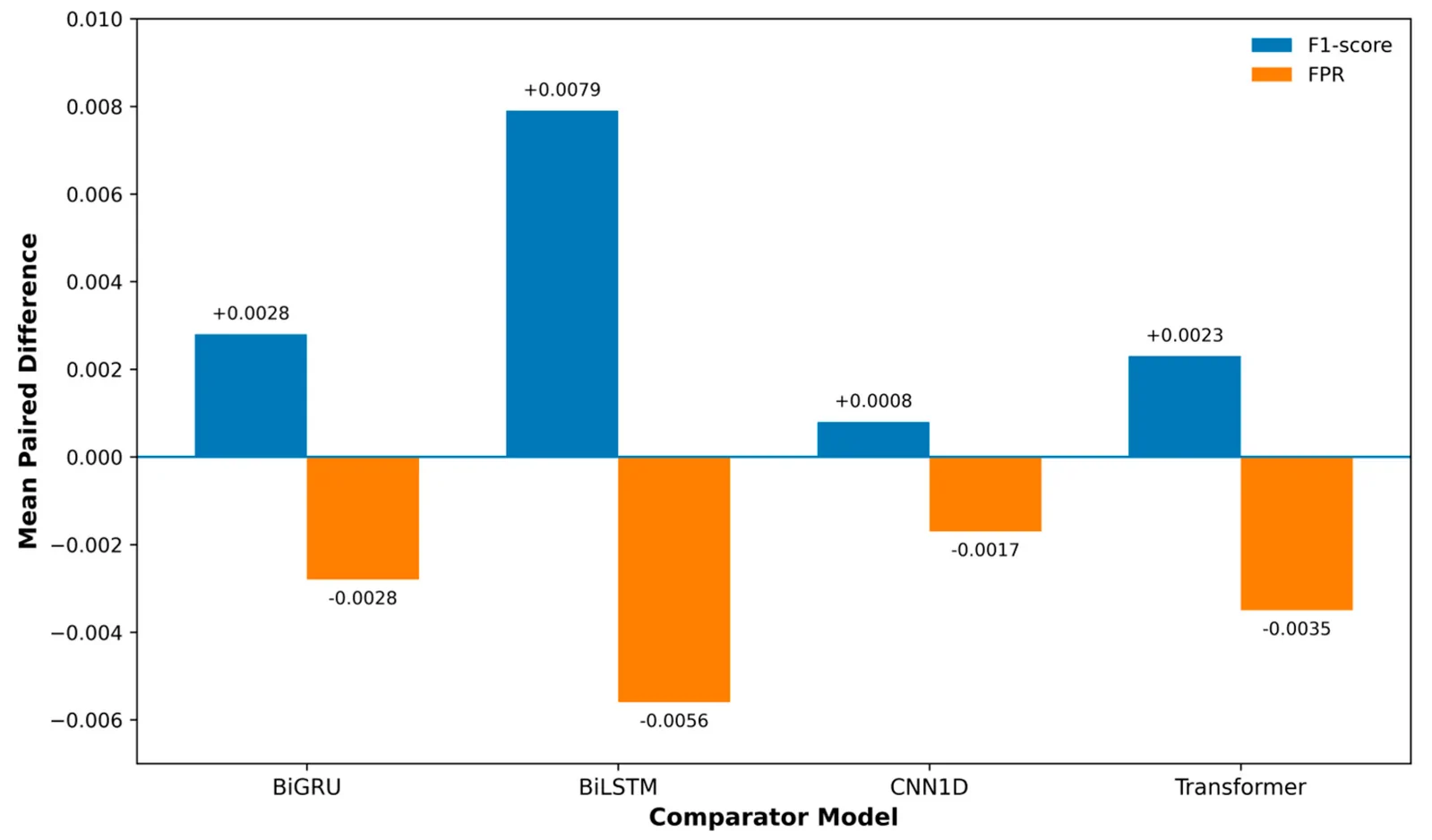
Mean paired differences in F1-score and FPR across the comparator models.

Holm–Bonferroni correction was applied to the four pairwise comparisons. The raw *p*-values were ranked from smallest to largest, adjusted using the corresponding Holm factors, and then constrained to remain monotonic. After correction, all four comparisons had a *p*-value of 0.1252 as further illustrated in Figure 7.

### 4.5. Ablation Study

Table 5 evaluates the contribution of the convolutional, recurrent, and attention components. The complete architecture achieves an F1-score of 0.9997 ± 0.0004, matching the CNN–BiGRU variant at the reported precision. CNN1D, CNN–Attention, and BiGRU–Attention achieve F1-scores of 0.9988 ± 0.0006, 0.9983 ± 0.0008, and 0.9980 ± 0.0012, respectively. The complete model improves the F1-score by 0.0017 compared with the weakest ablation variant, BiGRU–Attention (0.9980), which represents a relative gain of approximately 0.17%. However, based on the corrected *p*-values reported in Table 5, this difference is not statistically significant at conventional significance levels. The CNN1D variant contains substantially fewer parameters at 58,753. The ablation results show that the CNN–BiGRU configuration already provides very high detection performance on the benchmark. The complete architecture and the CNN–BiGRU variant have the highest observed F1-score, although their reported means are identical. The corrected F1-score comparisons in Table 5 provide the statistical basis for interpreting differences between the complete architecture and the evaluated ablation variants.

### 4.6. Explainability Analysis: SHAP and LIME

**Figure 8:**
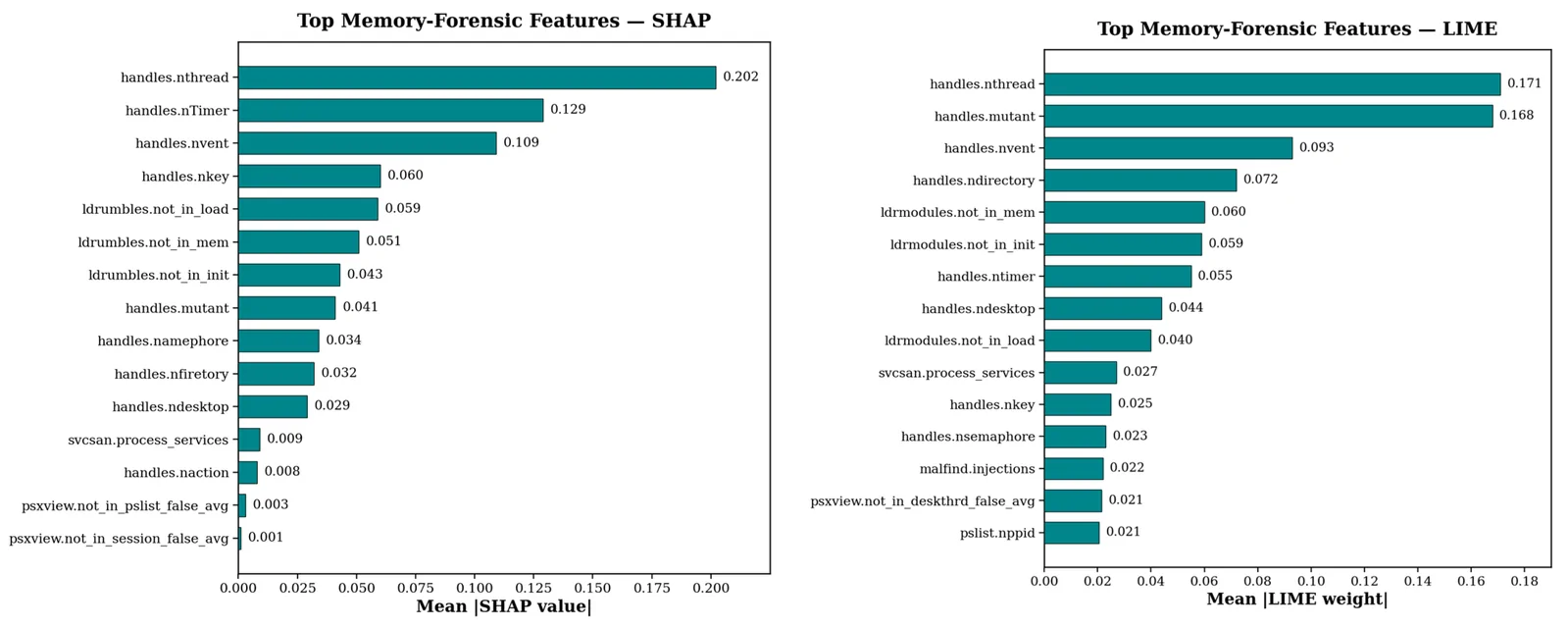
Global feature-importance comparison between SHAP and LIME.

Table 6 summarizes the quantitative comparison between SHAP and LIME using feature agreement, deletion sensitivity, runtime, and stability. The mean top-10 Jaccard overlap is 0.651 ± 0.136. Seven of the eight leading features are shared by the two methods. The handles.nthread feature is ranked first by both methods, while features from the handles and ldrmodules groups also appear prominently among the leading variables. As shown in Figure 8. The deletion analysis yields a mean change in the predicted probability of maliciousness of 0.362 for SHAP and 0.469 for LIME. These values measure sensitivity to the specified feature-removal operation and should not be interpreted as a standalone measure of explanation fidelity.

The explainers also differ in runtime. SHAP requires 7.96 s in total, or 0.080 s per sample, whereas LIME requires 35.38 s, or 0.354 s per sample. Under the evaluated configuration, SHAP was approximately 4.4 times faster. The reported top-10 stability was also higher for SHAP (0.888) than for LIME (0.731). The stability comparison should be interpreted with caution because the two methods were evaluated under different sources of randomness. SHAP used independent background-sample redraws, whereas LIME used independent explainer seeds. The comparison, therefore, provides directional evidence rather than a fully controlled assessment. The SHAP–LIME analysis was conducted on a fixed subset of 100 test instances. This subset was selected to keep the LIME perturbation procedure computationally manageable. Consequently, the reported agreement and stability values describe the evaluated subset rather than the entire test population.

### 4.7. Computational Efficiency

Table 7 compares the computational characteristics of the evaluated architectures using parameter count, model size, training time, inference latency, and throughput. All measurements were obtained under the same Google Colab environment with a Tesla T4 GPU.

**Figure 9:**
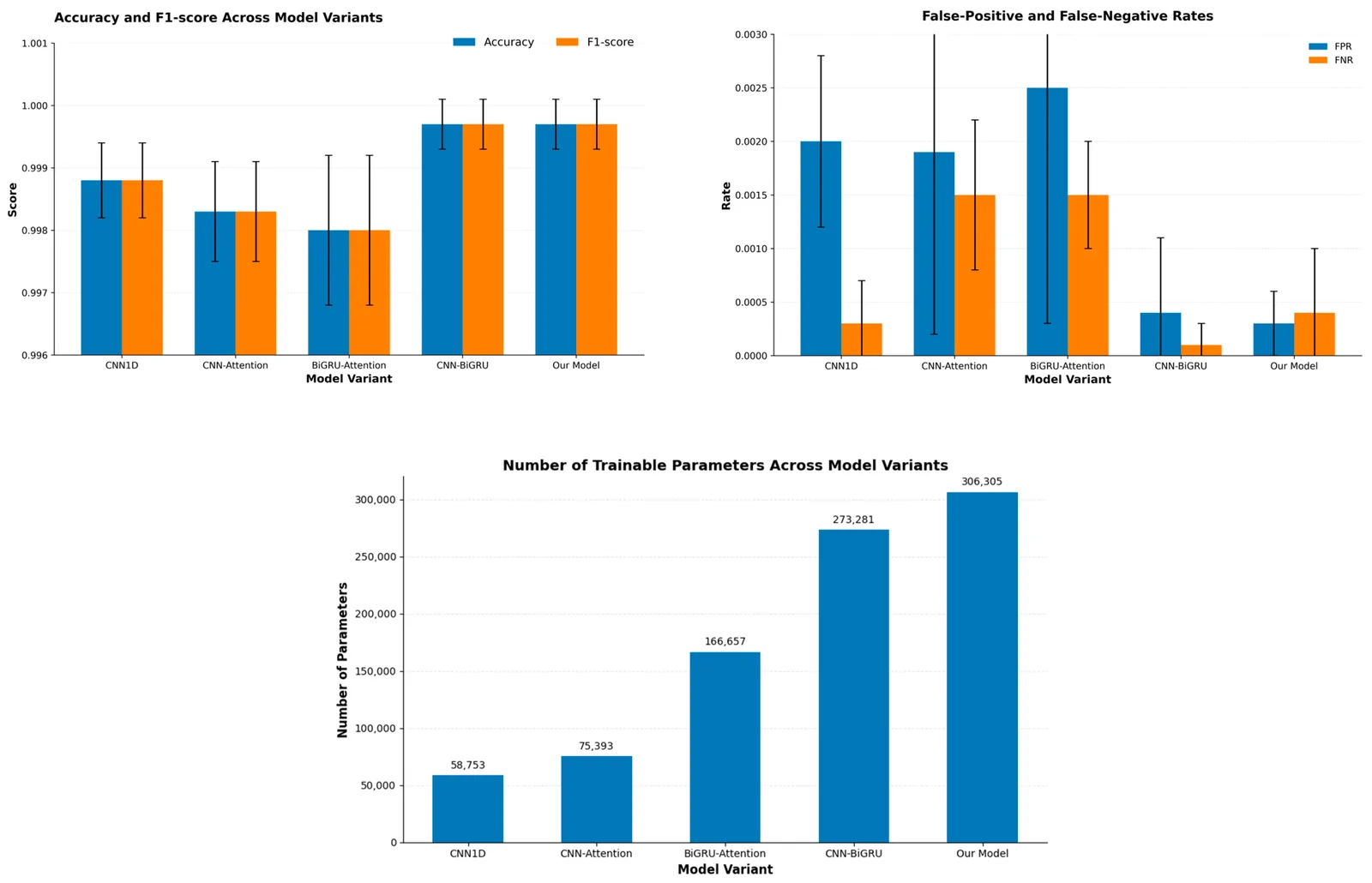
Computation efficiency visualization.

The proposed model has the largest parameter count (306,305) and model size (2.58 MB) as shown in Table 7 and Figure 9. It nevertheless records the shortest observed mean training time at 113.9 ± 21.7 s. CNN1D requires 149.9 ± 49.0 s, Transformer 167.7 ± 86.7 s, BiGRU 205.5 ± 82.9 s, and BiLSTM 133.3 ± 54.8 s. Training time depends on both model computation and the number of epochs completed before early stopping. The shorter wall-clock time of the proposed model should therefore not be interpreted as evidence that it has intrinsically lower optimization cost. For inference, the proposed model records a latency of 0.060 ± 0.003 ms per sample and a throughput of 12,661 ± 775 samples/s. CNN1D has the lowest latency, at 0.050 ± 0.008 ms per sample, and the highest throughput, at 20,477 ± 3227 samples/s. Relative to CNN1D, the proposed model has approximately 20% higher per-sample latency and approximately 38% lower throughput. These results demonstrate a measurable trade-off between detection performance and computational cost. The proposed architecture records the highest observed classification metrics, whereas CNN1D provides higher inference throughput.

### 4.8. Discussion

The proposed CNN–BiGRU model with additive attention achieved an F1-score of 0.9997 ± 0.0004 and MCC of 0.9993 ± 0.0007 on CIC-MalMem-2022. The small variation across the five training seeds indicates consistent behavior under the adopted protocol. However, the strongest architectures produced very similar results, and the corrected statistical analysis did not establish a statistically significant difference between the proposed model and the baseline architectures. The contribution of the complete architecture should therefore be interpreted in terms of its combined convolutional feature extraction, bidirectional recurrent processing, and attention, rather than as statistically demonstrated superiority. The error analysis provides additional information beyond aggregate accuracy. FPR and FNR remained close to zero, and the threshold analysis revealed a region where both error rates were low. These results are relevant to forensic screening, where both types of errors can affect subsequent investigation. The operating characteristics, however, were obtained on a single benchmark and should not be assumed to represent operational forensic environments. The SHAP–LIME analysis showed agreement in the leading feature rankings. SHAP required 7.96 s, compared with 35.38 s for LIME, under the evaluated configuration, and showed higher reported top-10 stability. These results indicate that post hoc attribution can provide feature-level information for forensic interpretation. The comparison remains limited by the fixed 100-instance test subset and the different randomization procedures used by the two explainers. The computational analysis revealed a clear trade-off. The proposed model had the largest parameter count and model size, while CNN1D achieved lower inference latency and higher throughput. The proposed model nevertheless recorded the shortest observed training time under the adopted early-stopping procedure. This should not be interpreted as a lower intrinsic optimization cost because training time also depends on the number of epochs completed. The study has several limitations. It uses a single benchmark dataset and a binary classification task, relies on VolMemLyzer for feature extraction, and evaluates SHAP and LIME on 100 test instances. In addition, the CNN and recurrent components process features in their original column order, so sensitivity to alternative feature orderings remains unevaluated. The near-ceiling performance of several models also limits the separation between architectures on this benchmark. Validation on independent memory-forensics datasets, newer acquisition environments, and broader malware categories is therefore required.

## 5. Conclusions

This study evaluated a CNN–BiGRU architecture with additive attention for binary malware detection on the CIC-MalMem-2022 dataset. It compared the model with CNN1D, BiGRU, BiLSTM, and Transformer baselines under a matched experimental protocol. The proposed model achieved an F1-score of 0.9997 ± 0.0004 and an MCC of 0.9993 ± 0.0007, with low FPR and FNR across five training seeds. The strongest architectures performed near the upper end of the benchmark, and the corrected statistical analysis did not establish a statistically significant difference between the proposed model and the baselines. The study also examined explainability and computational cost. SHAP and LIME showed agreement in their leading feature rankings, while SHAP required approximately 4.4 times less runtime under the evaluated configuration. The proposed model had the largest parameter count and model size, whereas CNN1D provided lower inference latency and higher throughput. These results show that detection performance should be considered together with explanation behavior and computational requirements. The main contribution is the matched evaluation of detection performance, error behavior, architectural variants, explainability, and computational efficiency within a common experimental framework. The findings are limited to the CIC-MalMem-2022 benchmark, binary detection, and the evaluated experimental conditions. Future work should test the framework on independent and more challenging memory-forensics datasets, broader malware categories, and operational acquisition environments.

## Figures and Tables

**Table 1:** Comparison of related work on deep learning and machine learning for memory-based malware detection.

Authors	Algorithms	Datasets	Accuracy (%)	FPR and FNR	Computational Efficiency		
					Training Time (s)	Inference Latency (ms)	Memory Usage (MB)
Dener et al. [7]	LR, RF, DT, MLP, DFFNN, LSTM (Spark)	CIC-MalMem-2022	99.97	×	×	×	×
Shafin et al. [12]	CNN–BiLSTM (CompactCBL/RobustCBL)	CIC-MalMem-2022	99.98	×	×	×	×
Shah et al. [5]	LBP + GLCM features + CNN (RGB memory images)	Custom	97.01	×	×	×	×
Cevallos-Salas et al. [20]	Logistic Regression, DNN + SMOTE	CIC-MalMem-2022	100	×	×	×	×
Mezina and Burget [11]	Dilated CNN	CIC-MalMem-2022	99.00	×	×	×	×
Naeem et al. [23]	Deep-stacked CNN ensemble + XAI	CIC-MalMem-2022	99.80	×	×	×	×
Liu et al. [9]	ResNet-18 + attention–GRU	Custom memory	98.34	×	×	×	×
Zhang et al. [10]	CNN1D on raw PE fragments	Custom PE-in-memory	97.48	×	×	×	×
Alrawashdeh [14]	Textual-attention transformer	CIC-MalMem-2022	97.00	×	×	×	×
Mystakidis et al. [19]	TabNet + LGBM/XGB/HGB voting ensemble	Custom-dataset	99.97	×	×	×	×
Proposed model	CNN–BiGRU with additive attention	CIC-MalMem-2022	99.97	0.03/0.04	113.9 ± 21.7	0.060 ± 0.003	2.58

The Accuracy column reports the binary detection result when explicitly available. (×) indicates that the corresponding result was not reported. Abbreviations: LR, logistic regression; RF, random forest; DT, decision tree; MLP, multilayer perceptron; DFFNN, deep feed-forward neural network; LSTM, long short-term memory; CNN, convolutional neural network; CNN1D, one-dimensional convolutional neural network; BiLSTM, bidirectional long short-term memory; LBP, local binary pattern; GLCM, gray-level co-occurrence matrix; DNN, deep neural network; SMOTE, synthetic minority over-sampling technique; GRU, gated recurrent unit; XAI, explainable artificial intelligence; LGBM, LightGBM; XGB, XGBoost; HGB, histogram gradient boosting; PE, Portable Executable.

**Table 2:** Detection performance of the compared architectures (mean ± SD, (*n* = 5) seeds).

Model	Accuracy	Precision	Recall	F1-Score	ROC-AUC	FPR	FNR	MCC
CNN1D	0.9988 ± 0.0006	0.9980 ± 0.0008	0.9997 ± 0.0004	0.9988 ± 0.0006	1.0000 ± 0.0000	0.0020	0.0003	0.9977 ± 0.0011
BiGRU	0.9969 ± 0.0030	0.9969 ± 0.0025	0.9969 ± 0.0036	0.9969 ± 0.0030	0.9989 ± 0.0018	0.0031	0.0031	0.9938 ± 0.0061
BiLSTM	0.9918 ± 0.0004	0.9941 ± 0.0015	0.9895 ± 0.0015	0.9918 ± 0.0004	0.9955 ± 0.0025	0.0059	0.0105	0.9836 ± 0.0008
Transformer	0.9974 ± 0.0007	0.9962 ± 0.0013	0.9985 ± 0.0007	0.9974 ± 0.0007	1.0000 ± 0.0000	0.0038	0.0015	0.9947 ± 0.0013
Our Model	0.9997 ± 0.0004	0.9997 ± 0.0003	0.9996 ± 0.0006	0.9997 ± 0.0004	1.0000 ± 0.0000	0.0003	0.0004	0.9993 ± 0.0007

**Table 3:** Default and validation-tuned thresholds for the proposed model across the five seeds.

Seed	Threshold	Default FPR	Default FNR	Tuned FPR	Tuned FNR
42	0.11	0.0000	0.00023	0.00091	0.00023
52	0.32	0.00046	0.00137	0.00091	0.00068
62	0.51	0.00046	0.0000	0.00046	0.0000
72	0.43	0.00068	0.00023	0.00114	0.00023
82	0.32	0.0000	0.0000	0.00046	0.0000

**Table 5:** Ablation results for the evaluated architectural variants.

Variant	Accuracy	F1-Score	FPR	FNR	Parameters	Holm-Corrected *p* (F1-Score)
CNN1D	0.9988 ± 0.0006	0.9988 ± 0.0006	0.0020 ± 0.0008	0.0003 ± 0.0004	58,753	0.1252
CNN–Attention	0.9983 ± 0.0008	0.9983 ± 0.0008	0.0019 ± 0.0017	0.0015 ± 0.0007	75,393	0.1252
BiGRU–Attention	0.9980 ± 0.0012	0.9980 ± 0.0012	0.0025 ± 0.0022	0.0015 ± 0.0005	166,657	0.1252
CNN–BiGRU	0.9997 ± 0.0004	0.9997 ± 0.0004	0.0004 ± 0.0007	0.0001 ± 0.0002	273,281	0.1252
Our Model	0.9997 ± 0.0004	0.9997 ± 0.0004	0.0003 ± 0.0003	0.0004 ± 0.0006	306,305	—

Holm–Bonferroni correction was applied to the four pairwise comparisons. The raw *p*-values were ranked from smallest to largest, adjusted using the corresponding Holm factors, and then constrained to remain monotonic. After correction, all four comparisons had a *p*-value of 0.1252.

**Table 6:** Quantitative comparison of SHAP and LIME.

Measure	Value
Mean top-10 Jaccard overlap	0.651 ± 0.136
Mean deletion-induced probability change (Δp), SHAP	0.362
Mean deletion-induced probability change (Δp), LIME	0.469
Runtime, SHAP (total/per sample)	7.96 s/0.080 s
Runtime, LIME (total/per sample)	35.38 s/0.354 s
Top-10 stability, SHAP (independent background redraw, n = 20)	0.888
Top-10 stability, LIME (independent explainer seed, n = 20)	0.731

**Table 7:** Computational efficiency of the evaluated architectures (mean ± SD, (n = 5) seeds).

Model	Parameters	Model Size (MB)	Train Time (s)	Inference (ms/Sample)	Throughput (Samples/s)
CNN1D	58,753	0.72	149.9 ± 49.0	0.050 ± 0.008	20,477 ± 3227
Transformer	116,481	1.56	167.7 ± 86.7	0.056 ± 0.009	18,323 ± 3070
BiGRU	133,633	1.58	205.5 ± 82.9	0.073 ± 0.009	13,917 ± 1831
BiLSTM	166,145	1.95	133.3 ± 54.8	0.068 ± 0.007	14,748 ± 1545
Our Model	306,305	2.58	113.9 ± 21.7	0.060 ± 0.003	12,661 ± 775

## Data Availability

The original data presented in this study are openly available in the CIC-MalMem-2022 repository, maintained by the Canadian Institute for Cybersecurity at the University of New Brunswick, at https://www.unb.ca/cic/datasets/malmem-2022.html (accessed on 23 May 2026).
